# Age-dependent decline in stress response capacity revealed by proteins dynamics analysis

**DOI:** 10.1038/s41598-020-72167-4

**Published:** 2020-09-16

**Authors:** Kaiyue Chen, Wenting Shen, Zhiwen Zhang, Fangzheng Xiong, Qi Ouyang, Chunxiong Luo

**Affiliations:** 1 The State Key Laboratory for Artificial Microstructures and Mesoscopic Physics, School of Physics, Peking University, Beijing, China; 2 Center for Quantitative Biology, Academy for Advanced Interdisciplinary Studies, Peking University, Beijing, China; 3 Peking-Tsinghua Center for Life Sciences, Peking University, Beijing, China

**Keywords:** Cell biology, Systems biology

## Abstract

The aging process is regarded as the progressive loss of physiological integrity, leading to impaired biological functions and the increased vulnerability to death. Among various biological functions, stress response capacity enables cells to alter gene expression patterns and survive when facing internal and external stresses. Here, we explored changes in stress response capacity during the replicative aging of *Saccharomyces cerevisiae*. To this end, we used a high-throughput microfluidic device to deliver intermittent pulses of osmotic stress and tracked the dynamic changes in the production of downstream stress-responsive proteins, in a large number of individual aging cells. Cells showed a gradual decline in stress response capacity of these osmotic-related downstream proteins during the aging process after the first 5 generations. Among the downstream stress-responsive genes and unrelated genes tested, the residual level of response capacity of Trehalose-6-Phosphate Synthase (TPS2) showed the best correlation with the cell remaining lifespan. By monitor dynamics of the upstream transcription factors and mRNA of *Tps2*, it was suggested that the decline in downstream stress response capacity was caused by the decline of translational rate of these proteins during aging.

## Introduction

Aging is an inevitable consequence of life, and it results in the decline of various physiological functions, eventually leading to the death of the organism^1^. Various model organisms are used to research aging, including budding yeast, *C. elegans*, *Drosophila melanogaster* and so on. Studies with these model organisms have provided deep insights into the mechanisms of aging. These studies have been instrumental in identifying genes^2–4^, uncovering signaling pathways^5,6^, and discovering chemical compounds shown to orchestrate a distinct set of cellular processes that define organismal longevity in eukaryotes^7–9^. Among the different aging model systems, the detailed knowledge of budding yeast and the powerful tools developed to study its physiology make it a typical ideal model organism to study the mechanisms involved in aging^10^. Usually, the replicative lifespan (RLS) of budding yeast is examined in yeast aging studies; RLS is defined as the number of daughter cells produced by the mother cell before senescence^11^. Some biological processes of budding yeast cells, such as cell growth, mating behaviors, and sporulation efficiency, have been studied in the context of aging research^12,13^. Nevertheless, among various physiological functions, responses to external stresses are necessary for all organisms to survive or to increase their fitness in a complex environment^14^. The detection of changes in response behaviors during aging is much more important than the detection of sporulation or mating behaviors; Aging and stress response are inextricably linked. In *C. elegans*, the heat shock response(HSR) declines precipitously in early adulthood coincident with the onset of reproductive maturity^15^. However, currently, even in budding yeast, it remains difficult to characterize dynamic behaviors of stress response during organismal aging.

In recent years, microfluidic methods have been widely used in budding yeast aging studies^16–18^. By physically trapping mother cells, the daughter cells can automatically flow out when the mother cell and the daughter cells separate; thus, the lifespan of the mother cell can easily be analyzed. Microfluidic devices allow for well-controlled microenvironments in studies investigating the biological processes of mother cells^19^. These devices can usually be combined with fluorescence microscopy to study the dynamics of important protein production during the cell aging process^20^. Here, we used a new high-throughput microfluidic device, an automatic cell-cycle marker based RLS measurement method and other quantitative biology technologies to study the dynamics of the hyperosmotic stress response in the replicative aging process at the single-cell level. Our results proved that the osmotic stress was a weak stress that could be explored and determined stress response capacity change during cellular aging while having little influence on the aging process. On the other hand, we verified that the stress response capacity of the osmotic-related downstream proteins were correlated with replicative lifespan after first 5 generations and may be used as aging indicators or predictors. Furthermore, the dynamics of the upstream transcription factors involved in the osmotic response and the mRNA of downstream protein were studied and found to be almost unchanged when face osmotic stress during cell aging, which suggested that the decline in downstream stress response capacity should caused by the decline of translational rate of these proteins during aging.

## Results

### Device design and yeast strain construction

**Figure 1:**
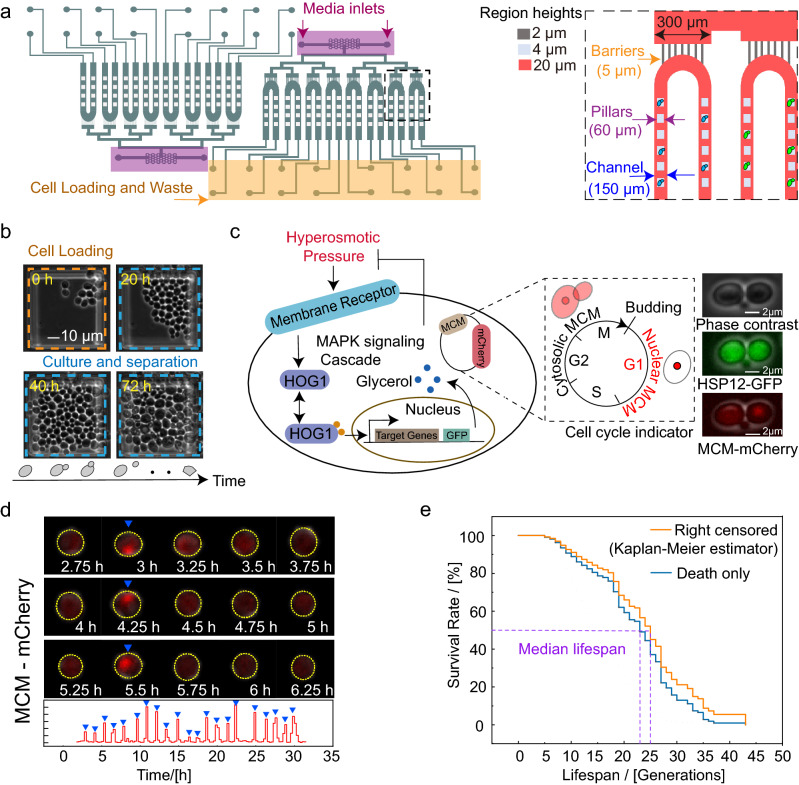
Principle of the microfluidic device and yeast strain construction. (**a**) (left) Schematic diagram of the overall image of the microfluidic chip. (right) Details of the barriers and cell traps. The chip was fabricated by three-layer structures with different heights for the barriers (2 µm), trapping pillars (4 µm) and the main channels (20 µm). Green and blue ellipses indicate yeast strains with different labels in different channels. (**b**) Images of yeast culture in a chip. Yeast cells were loaded into the chip, and mother cells were trapped and cultured under the pillar. (**c**) (left) Schematic representation of the HOG1-MAPK pathway and illustrative schematic of the dual reporter construct. The target genes were tagged with GFP, and the MCM gene associated with the cell cycle was labeled with m-Cherry. (right) Images of phase contrast, GFP and m-Cherry channels. The scale bar represents 2 µm. (**d**) Microscope images of the m-Cherry channel taken at the indicated time points during a part of the cell’s lifespan (up) and the nuclear m-cherry intensity of the cell as a function of time (down). The yellow contours highlight the cell of interest, and the blue triangles indicate the times at which budding occurred. (**e**) Kaplan–Meier estimator with right censored cells [orange curve] and cells born earlier than 15 h and died eventually during the observation time (blue curve). The intersections of the purple dotted line and the survival curves represents the median lifespan.

We designed a microfluidic device that can be used for high-throughput culture of different yeast strains under different specific environments and studied protein dynamics during cell aging at the single-cell level. The microfluidic device consists of two identical centrosymmetric units: one for the control experiment and the other for the stress response experiments (Fig. 1a, left). Each unit has eight parallel channel components for different yeast strains (strain channels) that have arrays of pensile columns (60 µm in size) inside the microfluidic channels that can physically trap mother cells in the gap between the column and the glass surface. The engineered height (4 μm) between the micropillars and the glass is similar to the diameter of yeast cells (4–5 μm), resulting in a single focal plane for long-term live-cell imaging. These parallel channels in the same unit are connected to two inlets by barrier structures with a 2-μm height, 5-μm width and 30-μm length to prevent the cross-contamination of multiple strains (Fig. 1a*,* right). The supplying culture media can be injected from the inlets and flow through the strain channels to wash away untrapped cells and to maintain a consistent microenvironment.

The protocol to load this chip is similar to the protocol described in our previous report work^21^. Cells of different strains are loaded into strain channels on the chip through the outlets using a pipette. Each strain channel is a U-channel where arrays of 80 pensile columns are located, ensuring that the trapped cells remain underneath the pensile columns. After loading the cells, the culture medium was injected into the inlets by computer-controlled syringe pumps. Time-lapse images of the trapped mother cells were obtained by both bright field and fluorescent microscopy with a Nikon Ti-E microscope. To validate the device, we confirmed that the majority of the loaded cells grew stably and exponentially under the micropillars (Fig. 1b).

To quantitatively measure general stress response and automatically recognize generations in the yeast aging process, we adopted a two-reporter gene approach (Fig. 1c). One reporter was GFP-labeled target genes downstream of high osmolarity glycerol Mitogen-activated protein kinase HOG1^22^. The other was the monomer-Cherry fluorescence protein (m-Cherry)-labeled cell cycle protein MCM. The MCM protein is known to enter the nucleus in G1 phase and to be exported from the nucleus during the S and M phases^23^. These strains were constructed by transferring a low-copy plasmid that contained the m-Cherry-tagged MCM gene into previously reported strains from the yeast GFP library.

### Automatic lifespan measurements under different microenvironments

In a systematic study, more than approximately one hundred cells are needed to estimate the lifespan of one strain under one condition; thus, it is difficult to manually collect this amount of data. Here, we developed an automatic method to record each generation and duration of each generation based on an MCM-mCherry reporter. To minimize the background influence, we defined the calculated index of the cell cycle (*I*_*c*_) as the following:

$$I_{c} = (I_{max} - I_{ave} )/I_{ave}$$

where *I*_*max*_ is the intracellular maximum fluorescence intensity and *I*_*ave*_ is the intracellular average fluorescence intensity.

As shown in Fig. 1d and Supplementary Movie, each *I*_*c*_ peak represents the cell entering the G1 phase, the distance between the two peaks is approximately equal to the duration of the cell cycle, and the number of peaks indicates the RLS of the cell. Therefore, we can easily obtain the duration and lifespan of many cells in experiments. The RLSs measured using our method (Fig. 1e) are comparable to those from similar microdissection methods using the manual counting method^24^.

**Figure 2:**
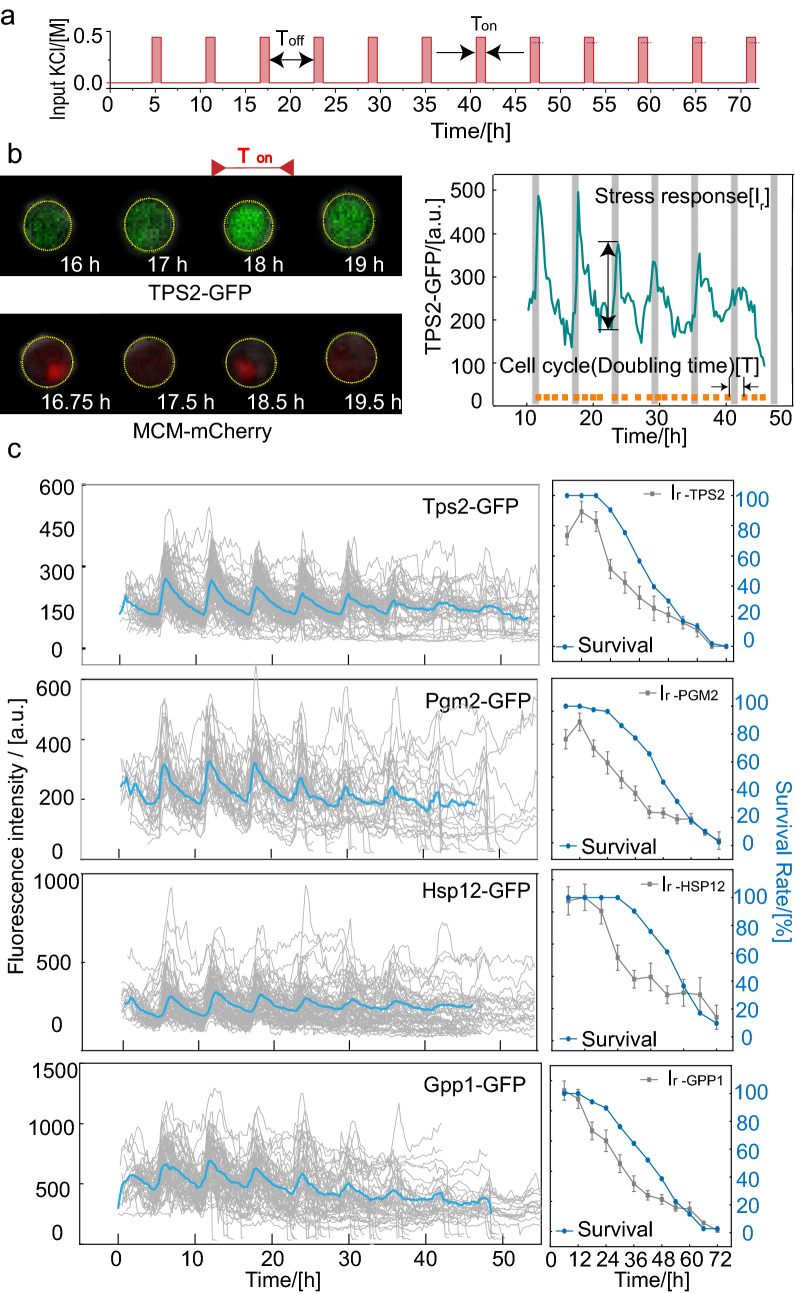
Yeasts undergo an age-induced successive decrease in stress response. (**a**) Osmotic signals considered in this work. *T*_*on*_ represents the time of the addition of the culture solution, which contained 0.4 M KCl, and *T*_*off*_ represents the time of the addition of the normal culture solution, where *T*_*on*_ = 1 h, and *T*_*off*_ = 5 h. (**b**) Sequence of green and m-cherry fluorescence images taken at the indicated time points. The yellow circles highlight the cell of interest, and magenta arrows represent the time of the stimuli (left). One representative trace of TPS2 in a single cell in the 0.4 M KCl stress experiment. The shaded area represents the time of the stimuli, and orange solid dots indicate the budding events. A schematic defines the stress response *I*_*r*_ of target proteins and doubling time T in a single-cell time trace (right). (**c**) Sequential protein level in the process of aging under periodic osmotic stimuli (left); The survival rate (blue dotted line) and osmosis-related stress response (gray dotted line and error bars) during the process of aging (right) (cell number N_TPS2_ = 108, N_HSP12_ = 42, N_PGM2_ = 79, N_GPP1_ = 67).

Osmoregulation is the active control of cellular water balance and encompasses homeostatic mechanisms required for life. In our preliminary experiments, we measured increases in the production of target proteins and in the recovery times of yeasts under sustained hyperosmotic pressure (0.4 M KCl) stimulation of approximately 1 h and 3 h (Supplementary Fig. S1). On the other hand, other work also show that the long term memory would thoroughly decay after osmotic stress within 4 h^25^. Therefore, we introduced periodic stimuli (1-h stimuli with 0.4 M KCl with 5 h periods of normal medium in between for 72 h) to facilitate the response data collection (Fig. 2a) and one single stimuli at time periods of 23–24 h as the control experiment.

To demonstrate that this periodic stimulation can be used to explore stress response during aging with little influence on lifespan, we measured the RLS of wild type strains under the two preset culture conditions (control condition and periodic stimuli) (Supplementary Fig. S2). The survival analysis of all mother cells showed that both conditions resulted in similar survival curves.

### Yeasts undergo an age-induced successive decrease in stress response

To investigate the dynamic changes in the osmotic stress response of aging yeasts, we monitored eight kinds of GFP-labeled target proteins, including four osmosis-related proteins, Trehalose-6-Phosphate Synthase (TPS2), Phosphoglucomutase (PGM2), Heat Shock Protein (HSP12), and Glycerol-3-Phosphate Phosphatase (GPP1), and four control proteins not involved in the osmotic stress response, Chromosome Transmission Fidelity (CTF4), Actin Patch Protein (APP1), Suppressor of Actin (SAC3), Stress-Seventy subfamily A(SSA2) in single cells using time-lapse fluorescence microscopy under a given osmotic condition (Fig. 2a and Supplementary Fig. S3). The proteins that correlated with osmotic stress response (components of the HOG1-MAPK pathway) exhibited periodic oscillations in response to the administration of periodic osmotic stimuli, whereas the proteins independent of osmotic stress did not show this production pattern (Fig. 2b and Supplementary Fig. S3). It was confirmed that the minimum and maximum synthesis rate of osmosis-related proteins during experiments were between the smallest and largest synthesis rates of the four control genes (Supplementary Table 1). Usually, osmosis-related genes were up/down-regulated after cells experience osmotic stress conditions^26^. After the removal of hyperosmotic stress, the levels of osmosis-related genes decreased markedly and approached the initial levels (Fig. 2b and Supplementary Fig. S3). To determine the change in stress response capacity during the aging process, we defined the difference between the peak after the stimuli and the base value of protein content as the stress response, *I*_*r*_, and the cell doubling time as *T* (Fig. 2b, right). We quantitatively measured the production level of the four proteins associated with osmotic stress response throughout the lifespan with periodical stimuli. The production level of all these proteins progressively decreased with cell age, following the trend in the survival rate (Fig. 2c). We found that the averaged response intensity of the four proteins showed a significant linear correlation with the corresponding survival rate after the third stimulation, and the best correlation coefficient was $R_{Tps2}^{2} = 0.93$(Supplementary Fig. S4).

### Quantitative analysis of stress response capacity at single-cell level and TPS2 is identified as the lifespan marker

**Figure 3:**
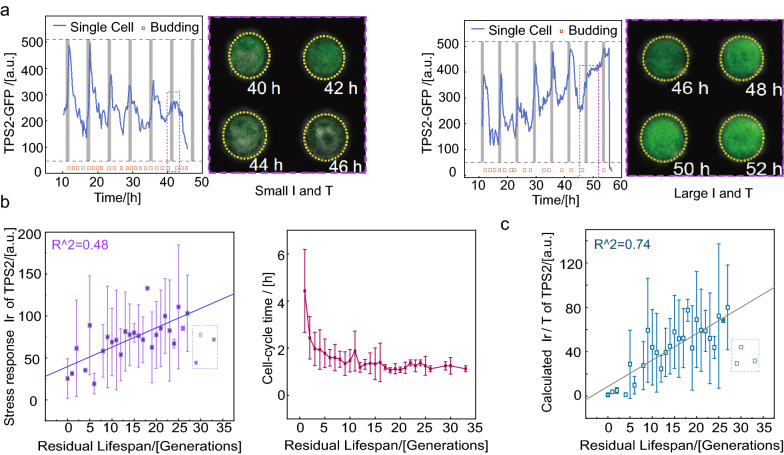
*I*_*r*_*/T* instead of *I*_*r*_ shows the better linear correlation with residual lifespan. (**a**) Two examples of GFP-labeled TPS2 before death in response to KCl stimuli. Vertical dashed lines indicate osmotic shock. (**b**) Relationship between averaged *I*_*r*_ and *T* of the TPS2-GFP strain and residual lifespan. The solid blue line represents the linear fitting line. In curve fitting, light data points in the dashed box are excluded. (**c**) Relationship between averaged *I*_*r*_*/T* of TPS2 and residual lifespan. The solid turquoise line represents the linear fitting line. In curve fitting, light data points in the dashed box are excluded.

Among the four stress response proteins, the response of TPS2 had the best correlation with the survival rate and production pattern under period osmotic pressure compared to three other proteins (Fig. 2c and Supplementary Fig. S4). Therefore, our subsequent analysis focused on the dynamic production of TPS2. In some cells with a relatively short cell cycle length, we observed that the TPS2-GFP response *I*_*r*_ eventually vanished at the moment of cell death; however, in other cells with a long cell cycle length, the TPS2-GFP response *I*_*r*_ remained at relative high levels (Fig. 3a). Therefore, when using *I*_*r*_ as a function of residual generations in single cell level unexpectedly only revealed a weak positive correlation (Fig. 3b, *R*^2^ = 0.48 exclude the data of residual generations more than 27).

On the other hand, we observed that the average cell cycle *T* underwent a “switch-like” transition from stable to an elongated cell cycle period and with enhance variation when the residual generations less than 5^27^ (Fig. 3b). Since the cells have two kinds of stress response modes near death may cause by the cell cycle duration difference(as shown in Fig. 3a), considering the influence of different dilution rates of cells (*1/T*), the stress response form (*I*_*r*_*/T*) of cells maybe more suitable to character the stress response capacity other than *I*_*r*_. As shown in Fig. 3c, *I*_*r*_*/T*, which we defined as the response capacity at the single-cell level as a function of residual generations, unexpectedly revealed a better positive correlation for many generations (*R*^2^ = 0.74).

Based on the above analysis, we can use the calculated *I*_*r*_*/T* as an indicator of lifespan expectancy because of the remarkable linear correlation between it and residual lifespan. It is worth noting that when the residual lifespan of the cells exceeds 27 generations, both *I*_*r*_ and *I*_*r*_*/T* significantly deviate, which may be due to the weak response capacity of the newborn cells when receiving stimulation. Therefore, we think that *I*_*r*_*/T* has excellent predictability when the residual lifespan is less than 27 generations.

**Figure 4:**
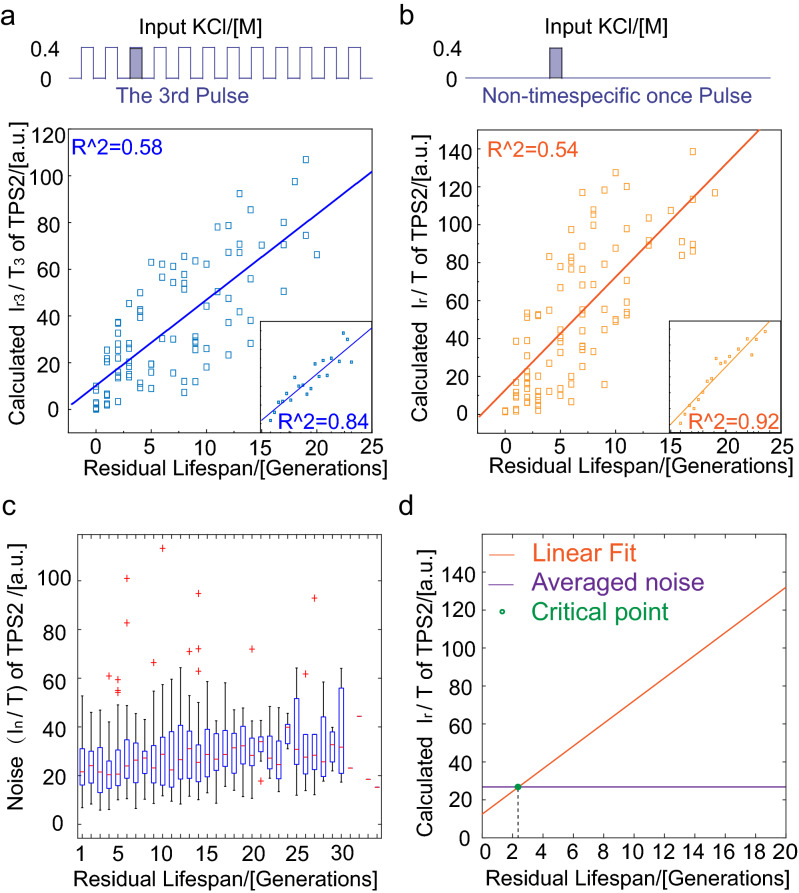
A weakening response in cellular stress response and TPS2 was identified as a lifespan marker. (**a**) Periods stimuli, every 6 h with 1-h stimuli of 0.4 M KCl for 72 h and (**b**) 1 h stimuli of 0.4 M KCl from 23 to 24 h. Response capacity *I*_*r*_*/T* of TPS2 and residual lifespan showed a significant positive correlation (two sets of experiments contained 97 and 99 cells, respectively). The lilac column represents the moment of adding osmotic stimulation. (**c**) Characterization of internal noise during the lifespan of yeast cells. (**d**) The relationship between TPS2 production (solid line, linear fit) and the internal noise (more than 60 cells).

To determine the best indicator for the residual lifespan of the cell, we separated the *I*_*r*_*/T* data by the stimulus times that the cells were exposed to after they were born. By analyzing the ability of four proteins in predicting residual lifespan at single-cell level (Supplementary Fig. S5), we found a striking linear positive correlation (*R*^2^ = 0.58 in single cell and *R*^2^ = 0.84 in average) between *I*_*r3*_*/T*_*3*_ (the ratio of the response intensity for the third stimulation to the cell cycle and the time of the third stimulation) of TPS2 and residual lifespan (Fig. 4a). This correlation remained when we administered one stress signal (Fig. 4b, *R*^2^ = 0.54, in single cell and *R*^2^ = 0.92 in average). Therefore, we can consider the stress response capacity of TPS2 as a clear hallmark of cell senescence; yeasts with larger *I*_*r*_*/T* lived considerably longer than those with smaller *I*_*r*_*/T*.

To explain why the protein production capacity can be considered as a residual lifespan marker, we used the simple comparison between stress response capacities *I*_*r*_*/T* with the internal noise during the whole lifespan. The internal noise *I*_*n*_*/T* hardly changed during the whole yeast cell lifespan. By comparing the fitting line of *I*_*r*_*/T* with the averaged internal noise *I*_*n*_*/T* (where *I*_*n*_ represents the difference between the maximum and minimum protein production levels in each cell cycle) throughout the lifespan (Fig. 4c), we found that the residual lifespan at the intersection of the two lines were about 2 generations (Fig. 4d), which suggested that the decreased accumulation of stress response proteins such as TPS2 maybe contribute to incompetent protection against internal noise fluctuation, which might result in cell death.

### Verification of the dynamics of transcription factors (TFs) and mRNA levels that remained unchanged during the aging process

**Figure 5:**
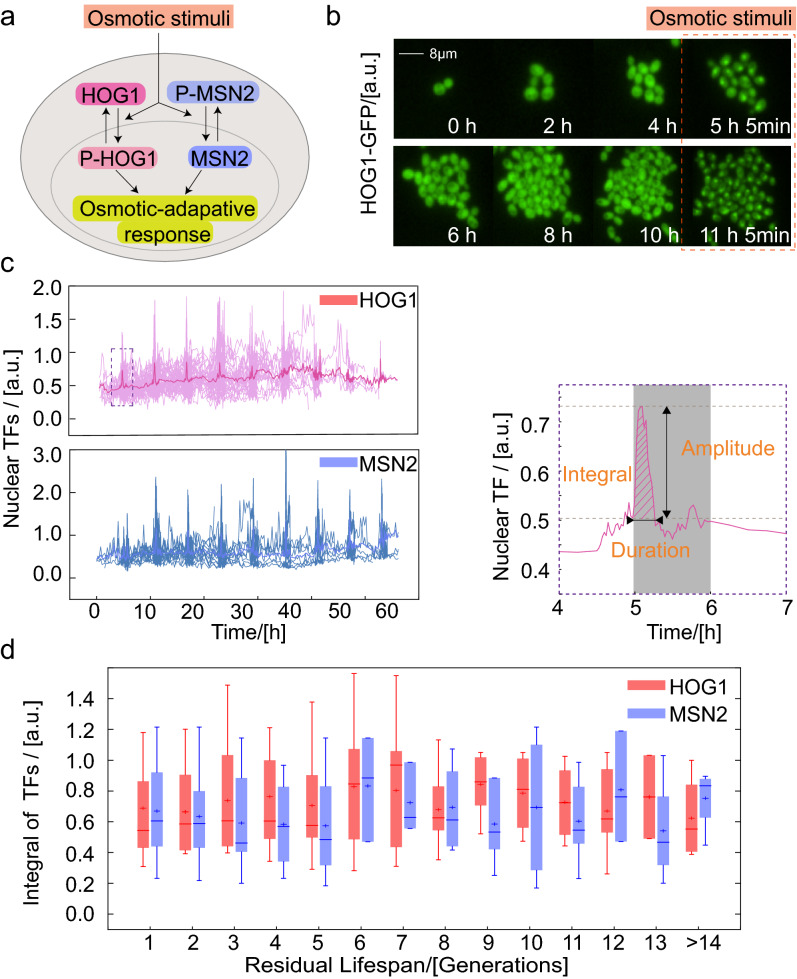
Transcription factors remain equivalent during the aging process. (**a**) Transcription factors (HOG1 and MSN2) translocate into the nucleus in response to osmotic stimuli. (**b**) HOG1-GFP images at the indicated times. The orange dotted box represents the stimulation time. (**c**) In response to a step stimulus of 0.4 M KCl, dynamic curves of single-cell HOG1 and MSN2 nuclear enrichment. Averaged trace of single-cells is shown in dark (left). The schematic defines the amplitude, duration and integral (oblique line) of the nuclear translocation of transcription factors in a single-cell time trace (right). (**d**) Integral of TFs corresponding to the datasets described in (**c**). Boxes extend from the 25th to 75th percentiles [interquartile range (IQR)]; horizontal lines represent the median, and whiskers indicate the lowest and highest datum within 1.5 × IQR from the lower and upper quartiles, respectively.

After finding that stress response capacity was reduced during aging, we next tried to investigate the regulatory mechanism of this process. The four kinds of osmotic-associated proteins are mainly regulated by two upstream TFs, HOG1 and MSN2 (Fig. 5a). Upon exposure to osmotic stimuli, HOG1 and MSN2 rapidly accumulate in the nucleus after being phosphorylated and dephosphorylated, respectively, where they bind to the promoter region of DNA and initiate the transcription of many downstream genes^28^. To reduce phototoxic damage to cells and accurately explore the nuclear localization process, we monitored the dynamic change in GFP-labeled TFs in the following two time periods during the aging process: cells were imaged at a short interval, every 3 min, when subjected to osmotic stimuli and at a long interval, every 15 min, when cultured under normal conditions. Changes in the localization of TFs was found (Fig. 5b), and the relative nuclear intensity was quantitatively computed as [TF(nucleus) − TF(cytoplasm)]/TF(cytoplasm) (Fig. 5c*,* left)^29^.

To evaluate the dynamics of TFs, we quantified the signals of GFP-labeled TFs by amplitude, duration and integral of the curve (Fig. 5c*,* right). For multiple TF translocation bursts in one individual cell under periodical osmotic stimuli, we observed a slightly increased baseline but no significant changes in amplitude and duration (Supplementary Fig. S6). Accordingly, the integral of HOG1 and MSN2 (above baseline) in the nucleus when facing osmotic stimuli was also stable as the residual generations of the cells decreased (Fig. 5d).

**Figure 6:**
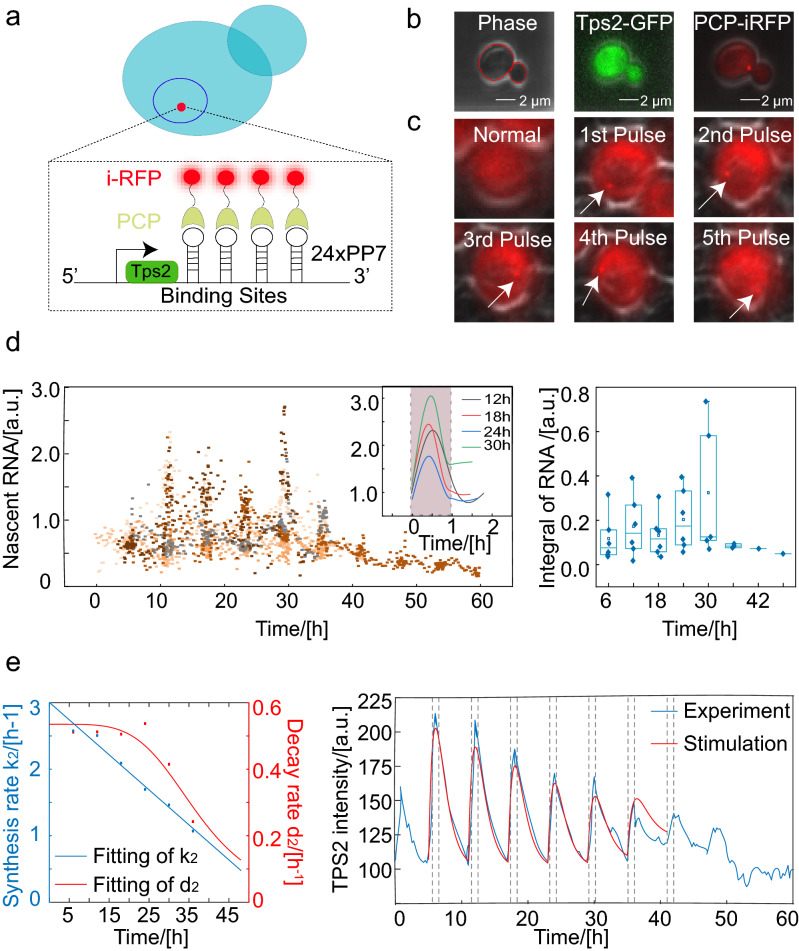
The weakening stress response during aging is mainly driven by decline of protein translation efficiency. (**a**) Design and characterization of the *Tps2-PP7-PCP* system. Schematic depicting the multiple stem-loop sequences (*PP7*) used to tag *Tps2,* and RNAs are visualized by the recruitment of iRFP-labeled PCPs. (**b**) Target protein and depiction of transcriptional bursting. Image of live cells coexpressing *Tps2-GFP* and *RNA-iRFP*. (**c**) Appearance of *Tps2* transcription site signals at the indicated times. White arrowheads denote transcription sites. (**d**) Transcriptional response to a sequence of stimulation pulses (six typical cells). Different colors represent different single cells. The inset is stress response patterns of RNA at different times during the aging process of one representative single cell. The shade represents the duration of stimulus application (left). Integral of mRNA calculated from six cell traces showed that the production of mRNA can maintain an almost stable value during the aging process (right). (**e**) (left) The change in the parameters during the cellular aging process and curve fitting. A linear function was used to fit k_2_, and a Hill function was used to fit d_2_. (right) Comparison of model fits to the experimental data sets for periodic 0.4 M KCl in budding yeast. The solid red and blue curves represent the theoretical and experimental results, respectively, and the vertical dashed lines indicate the start and end of the stimuli.

To further effectively decipher the mechanism of the dynamic change involved in the expression of downstream osmosis-related genes, we constructed a PP7-PCP platform for the real-time, single-cell detection of transcription in *Saccharomyces cerevisiae*^30^. The strain was constructed by inserting a cassette of 24 copies of *PP7* sequences into the 3′ UTR of the *Tps2* gene and recombining the *PCP-iRFP* gene segment to replace the original *lys2* gene. Thus, the nascent mRNA was bound by iRFP-labeled PP7-coated-proteins (PCP) shortly after transcription (Fig. 6a). Due to the accumulation of iRFPs at the transcription site, nascent RNAs can be detected as a fluorescent spot in single cells, allowing for their quantification (Fig. 6b). We induced *Tps2* transcription by periodically exposing the cells to a 0.4 M KCl signal, and imaged the cells at an interval of 3 min when the cells were under osmotic stress or at an interval of 15 min when the cells were cultured in normal media (Fig. 6c,d*,* left). To quantify the transcriptional dynamics of single cells, we defined the amplitude and duration of mRNA traces similar to TFs. After smoothing the mRNA transcription across the whole lifespan, we found that the response patterns of mRNA were very similar during the aging process. According to the single-cell traces and statistics, we can conclude that the transcriptional level (above baseline) is nearly invariable with age (Fig. 6d*,* right).

### Theoretical discussions of age-dependent decline in stress response capacity

To explore the reason for the gradual decline of TPS2 in stress response with cellular aging, we used a phenomenological model to analyze the dynamics of target proteins in response to periodic osmotic stress. Based on our experimental results, we assumed that the dynamics of TFs were the periodic square pulse (approximately 19 min) with a constant amplitude and duration (above baseline) to produce continuous time-dependent profiles that represented TF(t), the input for the following model:

1 $$TF\;(t) = \left\{ {\begin{array}{*{20}c} {TF_{0} ,} & {t > \Delta t} \\ {TF^{\prime},} & {0 < t < \Delta t} \\ \end{array} } \right.$$

where *Δt* is the duration of MSN2 or HOG1 nuclear localization.

The binding of a TF to DNA was assumed to be in equilibrium and governed by the following Hill equation: $\frac{{TF(t)^{n} }}{{K_{d}^{n} + TF(t)^{n} }}$. In our model, the binding of TFs immediately induced RNA production, which then lead to TPS2-GFP production. These processes are represented by the following coupled ordinary differential equations:

2 $$\frac{{d\left[ {mRNA} \right]}}{dt} = \frac{{k_{1} \cdot TF(t)^{n} }}{{K_{d}^{n} + TF(t)^{n} }} - d_{1} \times \left[ {mRNA} \right]$$

3 $$\frac{dP(t)}{{dt}} = k_{2} [mRNA] - d_{2} \times P(t)$$

*k*_1_ and *k*_2_ are the production rate of mRNA and protein; *d*_1_ and *d*_2_ are the mRNA and protein decay rates (degradation and dilution rate), respectively, and we considered *k*_1_ and *k*_2_ to be much larger than *d*_1_ and *d*_2_. The resulting average single-cell transcriptional trace was fitted with the exponential function, and *k*_1_ and *d*_1_ in our model were determined and almost keep constant during aging. The range of the mRNA decay rate *d*_1_ was determined to be about 3.0 h^−1^, comparable to a previous report^31^. The timescale of the production and decay of mRNA (~ 0.1 to 0.3 h) was much smaller than the timescale of the cell cycle (~ 1.5 h). Therefore, for the transcription process, the effect of growth dilution can be ignored, and the dynamics of *d*_1_ depends only on the degradation of mRNA. In addition, in late of stimuli (1 h), the concentration of protein almost reached a maximum concentration *P*_*max*_ as sustained hyperosmotic pressure (Supplementary Fig. S1). And we supposed that the mRNA level was *m*_1_ when protein reached the maximum concentration. So, in each stimulation cycle, we can get the following equations:

4 $$\frac{dP(t)}{{dt}} = k_{2} \times m_{0} - d_{2} \times P_{0} = 0$$

5 $$\frac{dP(t)}{{dt}} = k_{2} \times m_{1} - d_{2} \times P_{\max } = 0$$

6 $$d_{2} = \frac{{\ln \frac{{V_{T} }}{{V_{0} }}}}{T} + d_{2}^{\prime }$$

where *m*_0_ and *P*_0_ are the concentration of mRNA and protein at the start of a 1-h stimulation; *m*_*1*_ and *P*_*max*_ are the concentration of mRNA and protein when protein reach a maximum concentration after a 1-h stimulation.* V*_*T*_ is the total volume of the mother cell and the daughter cell when both cells enter the G1 phase, and *V*_0_ is the volume of the mother cell at the start of G1 phase. By fitting the data of each stimulation cycle in sections, we can determine the change in the protein decay rate *d*_2_ during aging. The variable *d*_2_ consists of the following two parts: the degradation rate $d_{2}^{\prime }$ and the dilution rate. We can separate the contributions of dilution and degradation based on experimentally measured changes in the cell cycle (Supplementary Fig. S7), and find that *d*_*2*_ is mainly caused by the dilution rate. As a consequence, *k*_*2*_ can also be fit to the experimental data, (Fig. 6e, left). Simulation models reproduced the protein production levels measured in response to period osmotic stress with the previous discussed parameters (Fig. 6e, right; Supplementary Fig. S8 and Supplementary Table 2).

According to Eqs. (4) and (5), the approximate relationships are *k*_*2*_ × *m*_*0*_ = *d*_*2*_ × *P*_*0*_ and *k*_*2*_ × *m*_*1*_ = *d*_*2*_ × *P*_*max.*_ Therefore, we found that the stress response capacity *I*_*r*_*/T,* which is proportional to *d*_*2*_ × (*P*_*max*_* − P*_*0*_), is also proportional to *k*_*2*_ × (*m*_1_* − m*_0_). Since the shape of mRNA dynamic curves were similar during cell aging, so the decline in *I*_*r*_*/T* during the aging process suggested that the decline in the translation rate *k*_*2*_ of stress-responsive proteins. Previous research has shown that bulk protein synthesis slows down during aging in a wide variety of organisms^32^ which supported our results. On the other hand, the transcription level of *Tps2* without stimuli showed slightly decline in the first 20 h (data no show), combined with the base level of TFs (HOG1 and MSN2) in nuclear and base level of mRNA increase in the first 20 h (Figs. 5c, 6d and Supplementary Fig. S8), which also suggested the decline of synthesis rate *k*_*2*_ of TPS2 at the early aging state with normal cell cycle duration.

## Discussion

We found that *S. cerevisiae* cells undergo a gradual decrease in response capacity to osmotic stress, but the upstream TF and mRNA level changed very little during the aging process. The utilization of a single-cell readout of transcription and translation enabled us to directly analyze the possible mechanism that the decrease in downstream protein production levels is modulated by posttranscriptional activity. Multiple studies demonstrated that global protein synthesis generally declines with increased organismal age^33^. Analyses of protein synthesis during aging to date have examined bulk protein synthesis not the translation of specific transcripts, so it’s unclear which proteins are being most affected^34^. We can utilize the microfluidic system to analyze the characteristics of specific proteins and conduct detailed research on whether other inducible proteins have the same results in future work. Our work also suggests that TPS2 response capacity is a highly predictive marker of yeast residual lifespan. Our studies are the first to reveal the quantitative relationship between the synthesis rate of TPS2 and residual lifespans and to provide an explanation for this predictability.

Further, the capability of this platform for high-throughput and precise environmental switching allows for the effective exploration of proteomics under different types (oxidative stress, nutritional deficiencies, etc.)^35,36^ and forms (ramp, oscillation, step, etc.)^37^ of stress in the aging of the yeast *S. cerevisiae*. Further work combining this method with genetic molecular mechanism studies will undoubtedly help to decipher the complex biological processes associated with replicative aging.

Recent studies have demonstrated that activation of the integrated stress response significantly contributes to lifespan extension in yeasts^38^. Most interventions that extend lifespan are, or induce, limited amounts of stress that have a beneficial effect via the phenomenon of hormesis. We hypothesize that combining inducible promoters, molecular intervention and microfluidics may be useful to manipulate the environment of cells to accurately change the protein production level of the stress-related genes and to prolong the lifespan of yeasts^39^. Importantly, since many of the components of longevity-regulating mechanisms are highly conserved from yeast to higher eukaryotes, the discovery of physiological regulators of these processes in yeast will likely have biological significance to human health and aging^40^.

## Methods

### Yeast strains and plasmid construction

The yeast strains used in this study were from the *S. cerevisiae* GFP fusion library generated by Dr. Erin O’Shea and Dr. Jonathan Weissman at UCSF, which includes 4,159 strains containing different proteins fused with the GFP protein from *Aequorea victoria* without affecting their functions.

The yeast strains were generated from the BY4741 (MAT a his3Δ1 leu2Δ0 met15Δ0 ura3Δ0) strain background. Yeast cells were prepared in synthetic defined media without the His and Ura amino acid complement and with 2% glucose. Yeast cell cultures were grown overnight to stationary phase and then allowed to resume exponential growth by dilution into fresh growth medium and incubation for 4–5 h at 30 °C before the experiment. The cells were then loaded into the microfluidic device. MCM-mCherry plasmid #(pCT05) from the Tang Chao Laboratory of Peking University^41^, was directly transformed into the yeast strain as a cell cycle marker.

### Microfluidic device fabrication

The microfluidic device fabrication protocol was similar to our previous work^21^. The mold for the microfluidic device was fabricated using a three-layer photolithography method to create SU8 photoresist (MicroChem, US) patterns with three heights on the silicon wafer. Afterwards, 6–8-mm thick PDMS was cast on the silicon wafers to transfer the shape of the designed structures. After curing at 70 °C for 3 h, the PDMS layer was peeled off the silicon wafer, and the inlets and outlets were punched. Then, the PDMS chip was bonded to 0.13 mm thick glass after oxygen plasma treatment and heated overnight at 70 °C. Before cell loading, the device was degassed in a vacuum for 20 min.

### Live-cell imaging and quantitative analysis of single-cell traces during aging

Time-lapse imaging experiments were performed using a Nikon Ti-E inverted fluorescence microscope with an EMCCD camera using a CFI S Plan Fluor ADM 40 × air objective (NA 0.60 WD 3.6–2.8 mm) or a CFI Plan Apochromat λ DM 60 × oil immersion objective (NA 1.40 WD 0.13 mm)^17^. The microfluidic device was placed in a 30 °C culture environment suitable for the growth of yeast cells and the yeast culture medium were provided by computer-controlled syringe pumps at the constant flow rate of 300 μl/h. Images were acquired every 15 min or at the designed periods for a total of 72 h.

We segmented the mother cell using phase images, which generated a mask to quantify the intensities of dual fluorescence reporters. To measure the green fluorescence of the labeled reporter genes, we quantified the mean fluorescence intensities of the whole cell due to their uniform distribution inside the cell. Cell nuclei or translation spots were identified by thresholding the m-Cherry or iRFP images. The mean value of the top 5 × 5 and 3 × 3 matrix pixels of m-Cherry and iRFP were used as the intensity of the cell cycle marker and mRNA indicator^42^.

### Replicative lifespan analysis

We used red fluorescent protein-labeled MCM to record the cell cycle. The MCM protein enters the nucleus in the G1 phase. Using the MATLAB program to find the peak of the nuclear MCM signal, the information describing cell generation was automatically obtained.

We counted cells which born in the initial to avoid collecting short live samples. After only counted the cells which born within the first 15 h and died within 72 h, the median lifespan was **22.8**. If incorporated all the cell which born within the firth 15 h but washed out before death into the lifespan curve as right-censored data, the median lifespan obtained was **25.0 (**Kaplan–Meier analysis) (Fig. 1e)^43^.

### *Tps2-PP7-PCP* system construction

The *Tps2-PCP-iRFP* strains were generated from the BY4741 (MAT a his3Δ1 leu2Δ0 met15Δ0 ura3Δ0) strain background. To construct the *Tps2-PCP-iRFP* strain, the GFP and 24XPP7 fragment was amplified by PCR and assembled by the Gibson method (#E2611—Gibson Assembly Master Mix). Then, the GFP-24XPP7 assembled fragment was amplified by PCR and integrated into the C-terminus of *Tps2* by homologous recombination. The 24XPP7 fragment was amplified by PCR from #(plasmid31864), and the RNA binding protein PCP-iRFP was amplified by PCR from #(pDZ276).

## Supplementary information

Supplementary Information 1.

Supplementary Information 2.

## Data Availability

The data that support the findings of this study are available on request from the corresponding author.
