# Supplementary material for: Age-dependent decline in stress response capacity revealed by proteins dynamics analysis
Source: Sci Rep. 2020 Sep 16;10:15211. doi: 10.1038/s41598-020-72167-4 (PMC7494919; doi:10.1038/s41598-020-72167-4)
Supplement: Supplementary file 1 — Supplementary Information 1. [file 41598_2020_72167_MOESM1_ESM.pdf]

Supplementary information for
Age-dependent decline in stress response capacity revealed by proteins dynamics analysis

Kaiyue Chen[1,2], Wenting Shen[2], Zhiwen Zhang[1], Fangzheng Xiong[1], Qi Ouyang[1,2,3], Chunxiong Luo[1,2]*

[1]The State Key Laboratory for Artificial Microstructures and Mesoscopic Physics, School of Physics, Peking University, China. [2]Center for Quantitative Biology, Academy for Advanced Interdisciplinary Studies, Peking University, China. [3]Peking-Tsinghua Center for Life Sciences, Peking University, Beijing, China.

*To whom correspondence should be addressed: Chunxiong Luo, email: pkuluocx@pku.edu.cn

Supplementary Figures

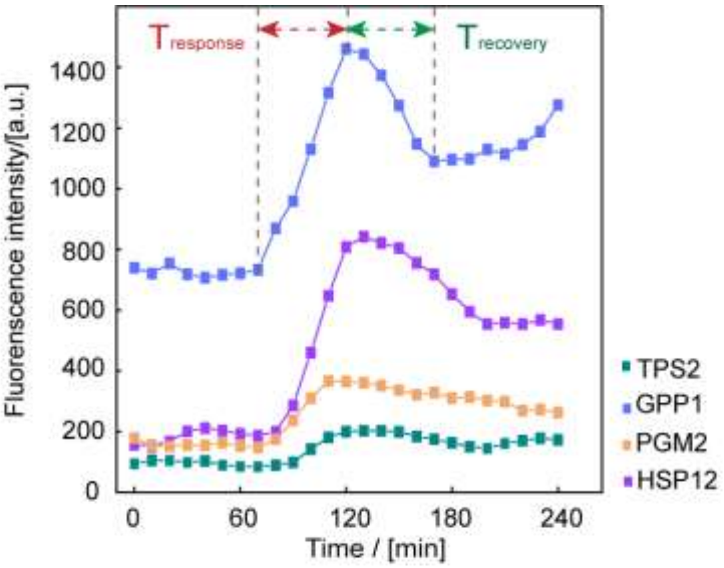

Supplementary Figure 1. Recovery times of yeasts under stimulation with sustained hyperosmotic pressure (0.4 M KCl).

To estimate the response and adaptation time of cells to osmotic stress, we added continuous osmotic pressure (0.4 M KCl) stimulation at 80 min. The response time is the time it takes for the base value to reach the fluorescence maximum. Recovery time is adapted from the maximum to the constant value. The response time and recovery time were approximately 1 h and 2~3 h, respectively.

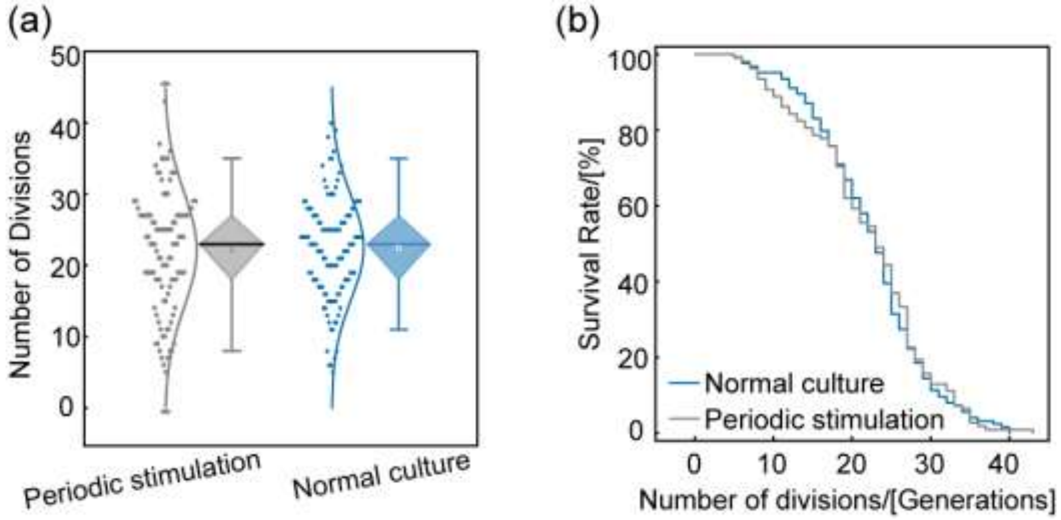

Supplementary Figure 2. Periodic mild osmotic stimulation (0.4 M KCl) does not affect the replicative lifespan of yeast cells.

(a) The lifespan distribution of yeast cells under normal culture and periodic stimulation conditions. The lifespan under both conditions showed a normal distribution, which is similar to previous conclusions obtained by using manual methods (N=124 and 108 under normal culture and periodic stimulation, respectively).

(b) Survival curves of yeast cells under two culture conditions. The replicative lifespan assay shows that compared to the normally cultured strain, strains that received the indicated stimulus exhibited a slight decrease in the mean number of generations.

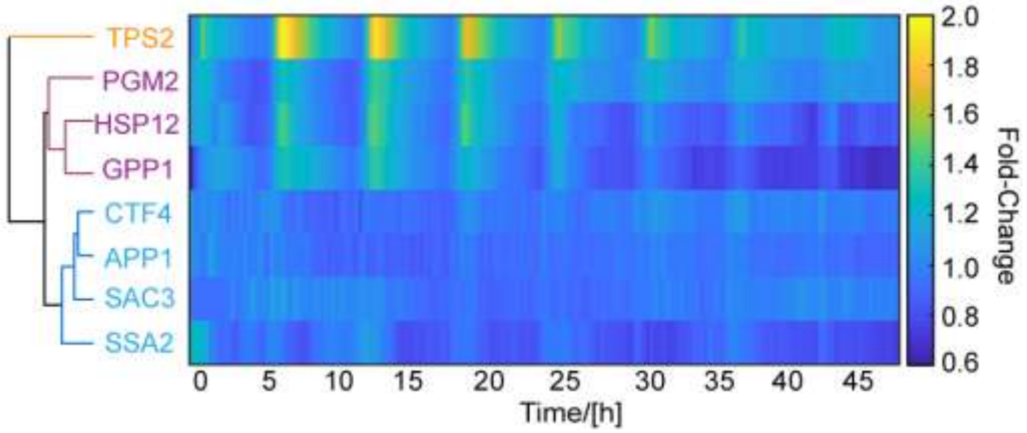

Supplementary Figure 3 Heat map depicts representative fold changes in the protein expression of components of the HOG1-MAPK pathway and housekeeping genes.

The colored bar represents the fold change in protein expression. Different categories are indicated with different colors. Osmotic-related proteins are marked in orange and purple, and the proteins independent of osmotic stress are in cyan.

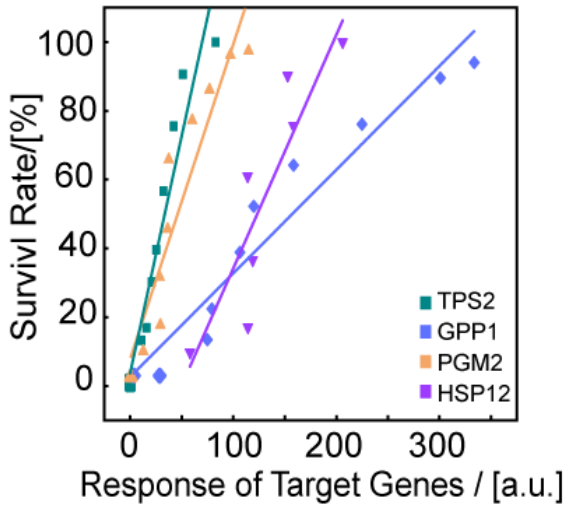

Supplementary Figure 4. Correlation analysis revealed a linear correlation between averaged stress responses.

For the four osmotic-related proteins, we performed a correlation analysis of the descending segments of the response curve and the survival curve. Correlation analysis revealed a linear correlation of higher than 80% on average between stress response and survival rate with the highest correlation coefficient of 93% in TPS2.

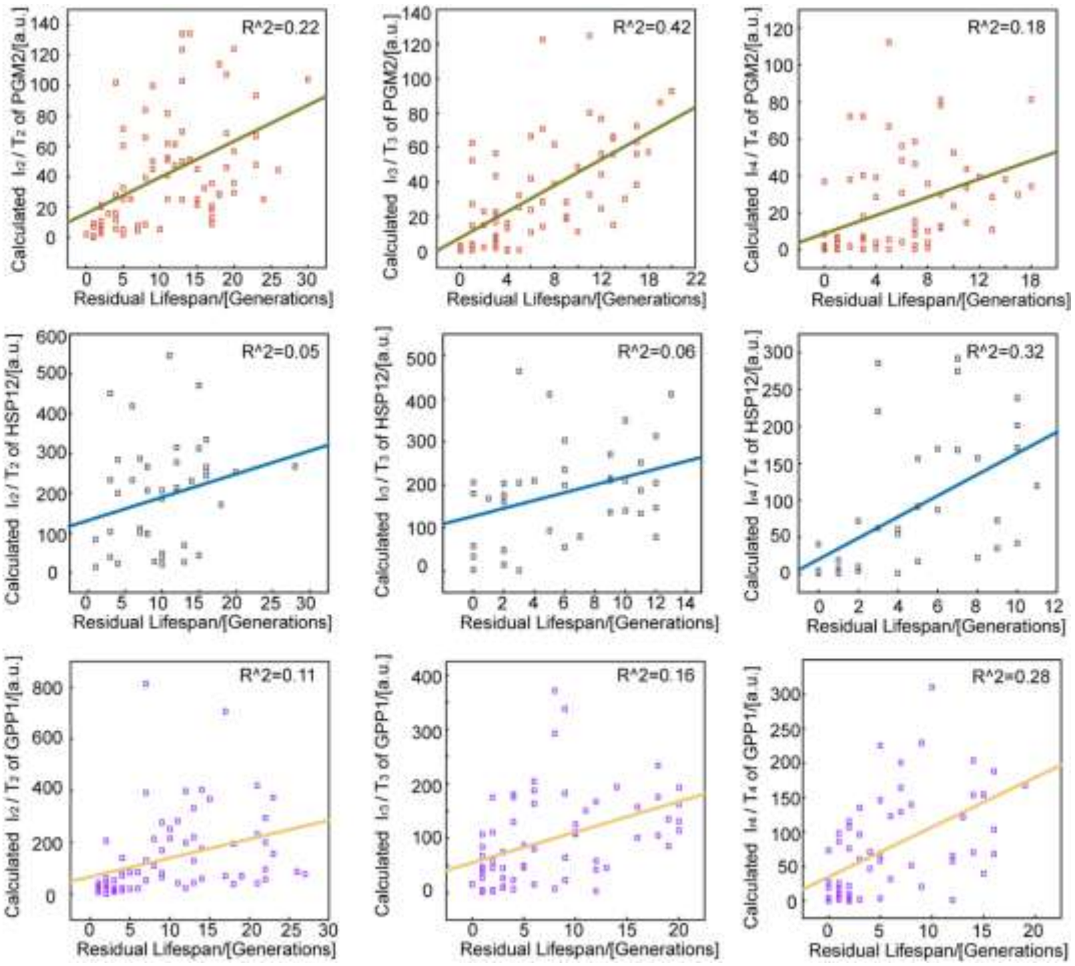

Supplementary Figure 5. Lifespan predictability of osmotic-related proteins.

We analyzed the relationship between the response capability of the four proteins to a single stimulus and the residual lifespan. Consistent with clustering results, TPS2 has the best lifespan in single-cell level (R^2=0.58, Fig. 4a), followed by PGM2 (R^2=0.42).

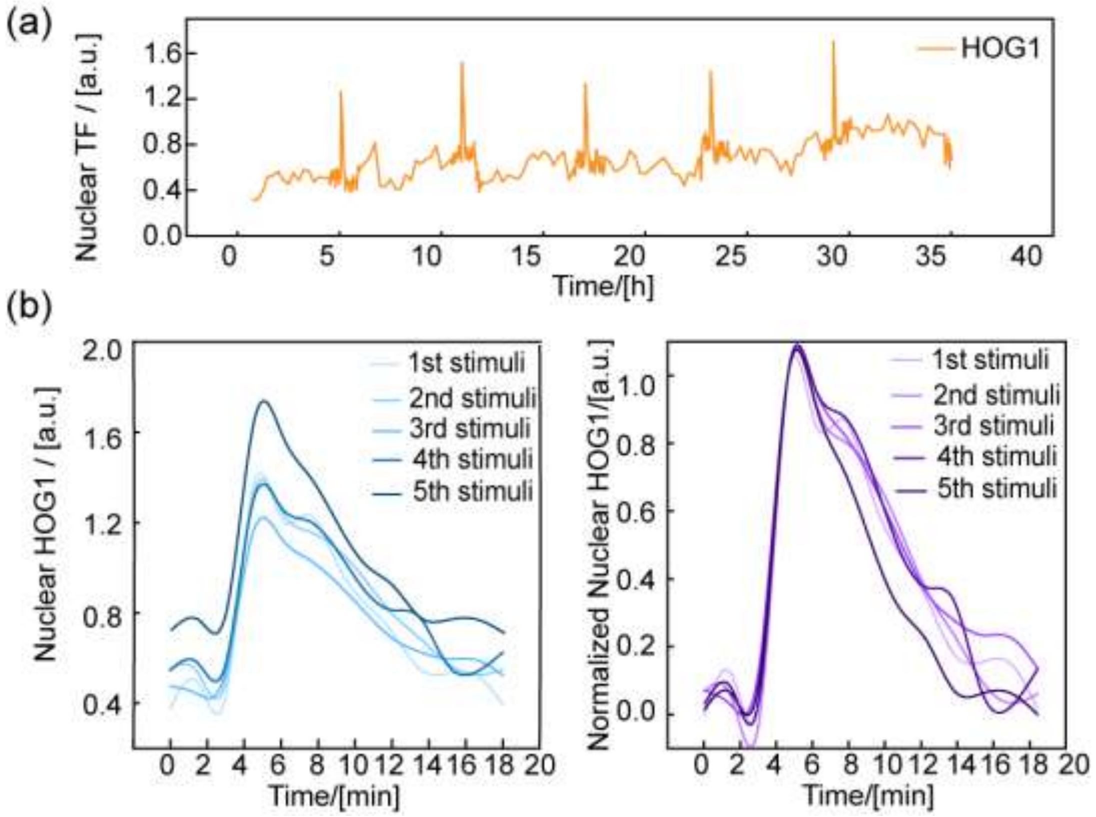

Supplementary Figure 6. Nuclear localization and duration analysis during aging at the single-cell level.

For single-cell data as an example (a), the fluorescent intensity and normalized nuclear HOG1 was demonstrated at each administered stimulation (b).

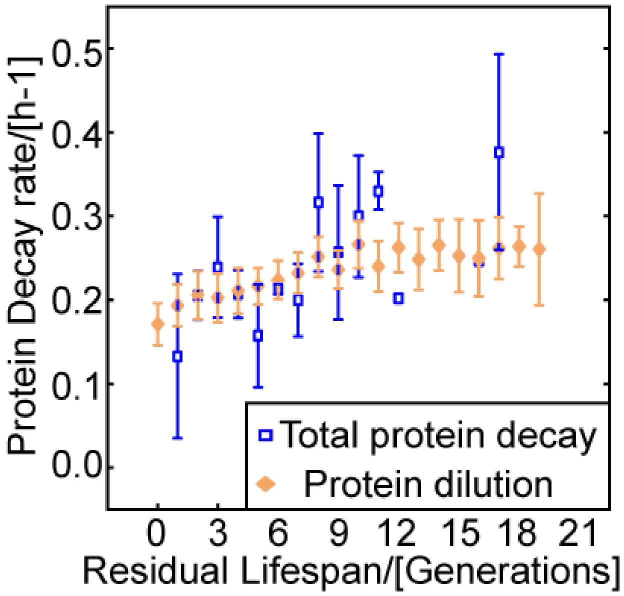

Supplementary Figure 7. Dynamic changes in protein dilution and degradation during the aging process.

We used single-cell data for parameter fitting to obtain the relationship between the decay coefficient of the target protein d_2 and residual generations (N=39). According to Equation (6), we quantitatively measured the protein dilution caused by cell division. Through the above analysis, we deduced the effect of molecular degradation on the remaining lifespan. Although the single-cell data are relatively messy, the degradation ability of the cells was significantly reduced in the generations before death.

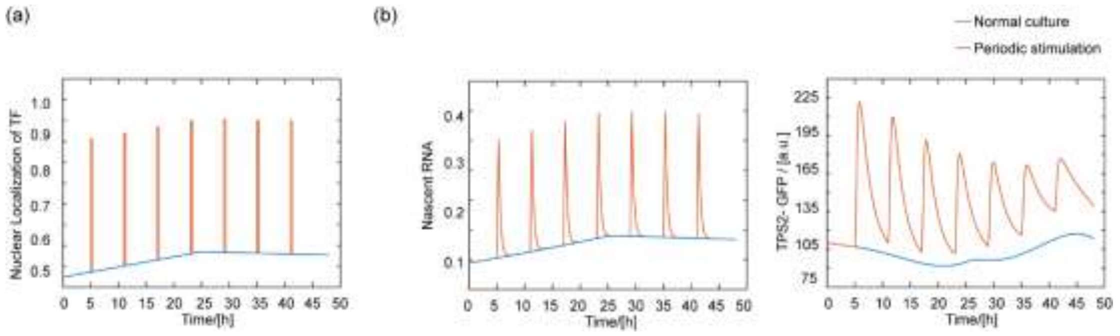

Supplementary Figure 8. Simulated time traces of mRNA and protein from different TF inputs.

(a)curve fitting of averaged transcription factor(TF) localization time trace throughout the whole lifespan.

(b)Simulations of mRNA and downstream protein expression in response to two different transcription factor(TF) inputs (orange solid line: periodic stimulation; blue solid line: normal culture).

Supplementary Table 1. Performance comparison of different abundance proteins.

	TPS2	PGM2	HSP12	GPP1	CTF4	APP1	SAC3	SSA2
copy numbers (untreated) （From Yeastgenome.org）	15818	40334	42197	221168	5044	1530	1677	411582
copy numbers (under stress) （From Yeastgenome.org）	31452	66625	84487	344606	NA	NA	NA	NA
Basic fluorescent value I_0 / [a.u.](Untreated)	≈ 100	≈ 200	≈ 200	≈500	≈ 40	≈ 30	≈ 30	≈ 1000
Maximum fluorescent value under osmotic-stress I_{max} / [a.u.]	≈ 220	≈ 300	≈ 400	≈700	≈ 45	≈ 40	≈ 40	≈ 1100
Maximum synthesis rate α_{max} ([a.u.]/h)	≈ 180	≈ 220	≈ 330	≈500	≈ 35	≈ 30	≈ 30	≈ 750
Averaged synthesis rate α_0 ([a.u.]/h)	≈ 80	≈ 170	≈ 170	≈350	≈ 30	≈ 25	≈ 25	≈ 700

NA means not analysed

I_0 and I_{max} are the results of our experiments.

α_{max} and α_0 are calculated by the Euler equation[1].

Computational modeling:

In this phenomenological model, the binding of transcription factor immediately induces mRNA production, which then leads to the production of downstream protein[2]. The equations describing the system as follows:

$$TF(t) = \begin{cases} TF_{0,} t > \Delta t \\ TF', 0 < t < \Delta t \end{cases}$$

$$\frac{d[mRNA]}{dt} = \frac{k_1 \cdot TF(t)^n}{K_d{}^n + TF(t)^n} - d_1 * [mRNA]$$

$$\frac{dP(t)}{dt} = k_2[mRNA] - d_2 * P(t)$$

$\boldsymbol{k_1}$ and $\boldsymbol{k_2}$ are the production rate of mRNA and protein; $\boldsymbol{d_1}$ and $\boldsymbol{d_2}$ are the mRNA and protein decay rates (degradation and dilution rate), respectively. The binding of transcription factor to DNA is governed by the Hill function, where $\boldsymbol{K_d}$ is the dissociation constant and $\boldsymbol{n}$ is the Hill

coefficient.Δt is the nuclear localization time of transcription factor.
Then, we can get the function of mRNA $m(t)$ and protein $P(t)$ through the ODE equations.

$$m(t) = \begin{cases} \frac{A}{d_1}(e^{d_1 t} - 1)e^{-d_1 t}, & 0 < t < \Delta t \\ \frac{A}{d_1}(e^{d_1 \Delta t} - 1)e^{-d_1 t}, & t > \Delta t \end{cases}$$

$$P(t) = \begin{cases} \frac{k_2 A}{d_2 - d_1}\left(\frac{1 - e^{-d_1 t}}{d_1} - \frac{1 - e^{-d_2 t}}{d_2}\right), & 0 < t < \Delta t \\ \frac{k_2 A}{d_2 - d_1}\left(\frac{e^{-d_1 t}(e^{d_1 \Delta t} - 1)}{d_1} - \frac{e^{-d_2 t}(e^{d_2 \Delta t} - 1)}{d_2}\right), & t > \Delta t \end{cases}$$

$$A = k_1\left(\frac{TF'^n}{K_d^n + TF'^n} - \frac{TF_0^n}{K_d^n + TF_0^n}\right)$$

We fitted the averaged protein and mRNA with the function $m(t)$ and $P(t)$.The parameter sets selected from the fitting are those that gave the smallest sun of squared residual(ssq) (Supplementary Table 2).

Supplementary Table 2. Parameter setting

$\boldsymbol{k_1}$	3.021 (h^{-1})
$\boldsymbol{d_1}$	3.0 (h^{-1})
$\boldsymbol{K_d}$	0.9232 (AU)
$\boldsymbol{n}$	4
$\boldsymbol{\Delta t}$	19 min

In order to verify the accuracy of the model, we fitted the time-series data of TF as input (Supplementary Fig. 8 a), and simulated the expression of mRNA and protein under given inputs (Supplementary Fig. 8 b). As shown in (Fig. 4c), the base value of transcription factors rises in the early lifespan and then flattens out, but the square pulses (above baseline) activated by osmotic stress does not change. For the two different transcription factor inputs (normal culture and periodic stimulation), the simulated protein dynamics trend is highly consistent with the experimental data.

Reference:

1. Zuleta IA, Aranda-Diaz A, Li H, El-Samad H. Dynamic characterization of growth and gene expression using high-throughput automated flow cytometry. *Nat Methods* **11**, 443-448 (2014).

2. Hao N, O'Shea EK. Signal-dependent dynamics of transcription factor translocation controls gene expression. *Nat Struct Mol Biol* **19**, 31-39 (2011).
